# Supplementary material for: Predictive Quantum Vibrational Spectra through Active Learning 4G-NNPs
Source: J Phys Chem Lett. 2026 Mar 5;17(11):3240–9. doi: 10.1021/acs.jpclett.5c03765 (PMC13007018; doi:10.1021/acs.jpclett.5c03765)
Supplement: Supplementary file 1 [file jz5c03765_si_001.pdf]

Supporting Information for "Predictive Quantum Vibrational Spectra through Active Learning 4G-NNPs"

Md Omar Faruque, Dil K. Limbu, Nathan London, and Mohammad R. Momeni*

Division of Energy, Matter and Systems, School of Science and Engineering, University of Missouri – Kansas City, Kansas City 64110, Missouri, United States

E-mail: mmomenitaheri@umkc.edu

Contents

Table S1. Parameters for the radial symmetry functions. S3

Table S2. Parameters for the angular symmetry functions. S4

Figure S1. Active learning benchmark. S5

Figure S2. Calculated correlation between the reference and 4G-HDCNNP-predicted charges, energies, and forces. S6

Figure S3. RDF comparisons between $n = 1 - 4$ committee models from classical MD simulations. S7

Table S3. Comparison of model errors with the literature. S8

Figure S4. Calculated correlation between the reference and final model predicted dipole moment for the bulk PIMD validation set. S9

Figure S5. Calculated correlation between the reference and final model predicted dipole moment of the air-water interface classical validation set. S10

Figure S6. Calculated correlation between the reference and final model predicted dipole moment of the air-water interface PIMD validation set. S11

Figure S7. RDF comparisons between simulation boxes with different numbers of bulk water molecules from classical MD simulations. S12

Figure S8. Comparison of Hann window cutoff. S13

Figure S9. IR spectra comparisons between 2G-HDCNNP and 4G-HDCNNP. . . . S14

Figure S10. Deconvolution of the O–H stretch peaks of the air-water interface from different PI methods. S15

Table S1: Parameters for the radial symmetry functions.

ID	Atom	Neighbor	η (1/Bohr^2)	R_s (Bohr)	R_c (Bohr)
1	H	H	0.001	0.0	12
2	H	H	0.010	0.0	12
3	H	H	0.030	0.0	12
4	H	H	0.060	0.0	12
5	H	H	0.150	1.9	12
6	H	H	0.300	1.9	12
7	H	H	0.600	1.9	12
8	H	H	1.500	1.9	12
9	H	O	0.001	0.0	12
10	H	O	0.010	0.0	12
11	H	O	0.030	0.0	12
12	H	O	0.060	0.0	12
13	H	O	0.150	0.9	12
14	H	O	0.300	0.9	12
15	H	O	0.600	0.9	12
16	H	O	1.500	0.9	12
17	O	H	0.001	0.0	12
18	O	H	0.010	0.0	12
19	O	H	0.030	0.0	12
20	O	H	0.060	0.0	12
21	O	H	0.150	0.9	12
22	O	H	0.300	0.9	12
23	O	H	0.600	0.9	12
24	O	H	1.500	0.9	12
25	O	O	0.001	0.0	12
26	O	O	0.010	0.0	12
27	O	O	0.030	0.0	12
28	O	O	0.060	0.0	12
29	O	O	0.150	4.0	12
30	O	O	0.300	4.0	12
31	O	O	0.600	4.0	12
32	O	O	1.500	4.0	12

Table S2: Parameters for the angular symmetry functions.

ID	Atom	Neighbor 1	Neighbor 2	η (1/Bohr^2)	λ	ζ	R_c (Bohr)
1	H	O	H	0.200	1.0	1.0	12
2	O	H	H	0.070	1.0	1.0	12
3	H	O	H	0.070	1.0	1.0	12
4	O	H	H	0.070	-1.0	1.0	12
5	H	O	H	0.070	-1.0	1.0	12
6	O	H	H	0.030	1.0	1.0	12
7	H	O	H	0.030	1.0	1.0	12
8	O	H	H	0.030	-1.0	1.0	12
9	H	O	H	0.030	-1.0	1.0	12
10	O	H	H	0.010	1.0	4.0	12
11	H	O	H	0.010	1.0	4.0	12
12	O	H	H	0.010	-1.0	4.0	12
13	H	O	H	0.010	-1.0	4.0	12
14	O	O	H	0.030	1.0	1.0	12
15	O	O	H	0.030	-1.0	1.0	12
16	O	O	H	0.001	1.0	4.0	12
17	O	O	H	0.001	-1.0	4.0	12
18	H	O	O	0.030	1.0	1.0	12
19	H	O	O	0.030	-1.0	1.0	12
20	H	O	O	0.001	1.0	4.0	12
21	H	O	O	0.001	-1.0	4.0	12
22	O	O	O	0.030	1.0	1.0	12
23	O	O	O	0.030	-1.0	1.0	12
24	O	O	O	0.001	1.0	4.0	12
25	O	O	O	0.001	-1.0	4.0	12

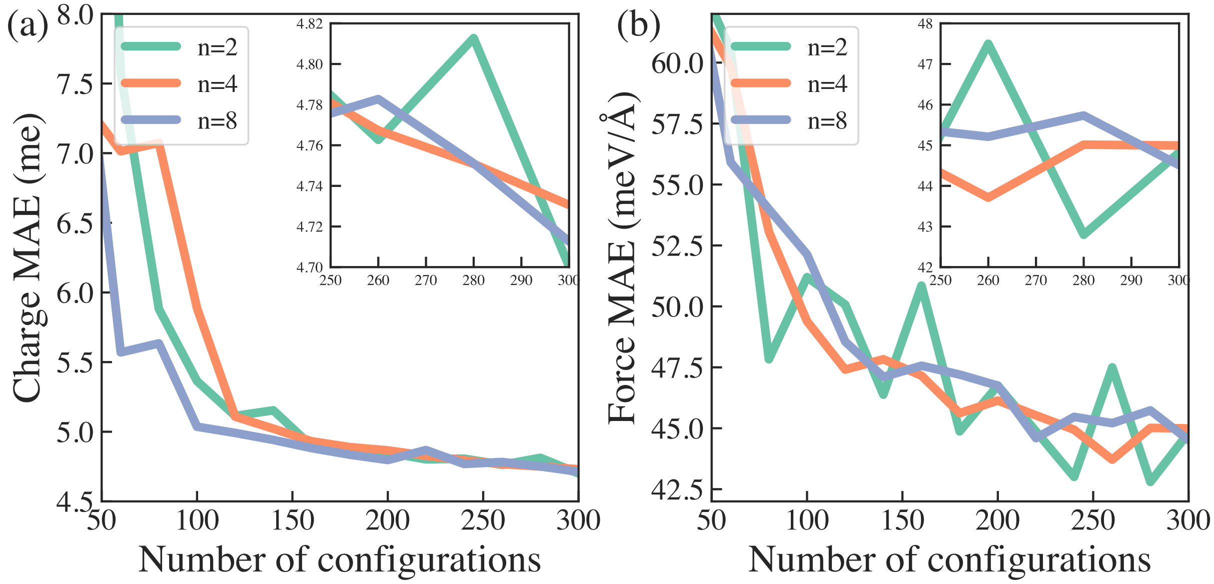

Figure S1: Active learning benchmark: (a) Charge MAE and (b) Force MAE for the number of models (n) in each committee.

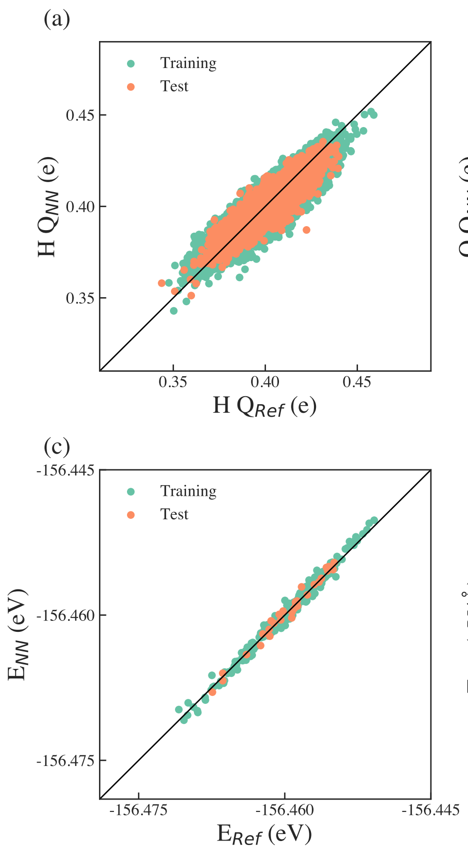

Figure S2: Calculated correlation between the reference and 4G-HDCNNP-predicted (a,b) charges, (c) energies, and (d) forces for both training and test sets.

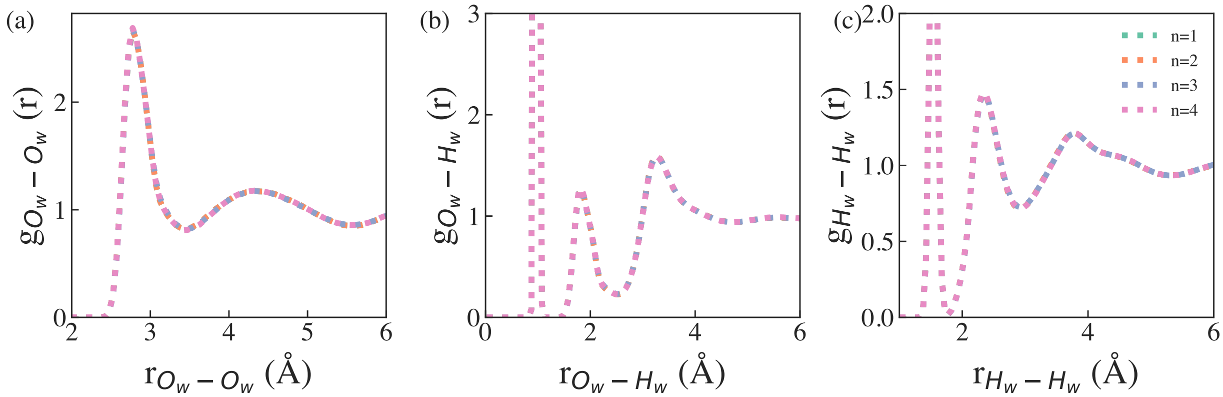

Figure S3: RDF comparisons between $n = 1 - 4$ committee models from classical MD simulations.

Table S3: Calculated RMSEs for the energy (meV/atom), force (eV/Å), and charges (me) of both the training and test sets of the committee 4G-HDNNPs developed in this work compared to data from the literature.

	**Train RMSE**	**Test RMSE**
**Q (me)**		
This work	5.97	5.91
Kocer et al.[1]	2.90	2.90
Ko et al.[2]	15.87	15.83
**E (meV/atom)**		
This work	0.45	0.41
Kocer et al.[1]	0.20	0.21
Ko et al.[2]	0.47	0.48
Schran et al.[3] (2G)	0.76	-
**F (eV/Å)**		
This work	0.05	0.05
Kocer et al.[1]	0.06	0.06
Ko et al.[2]	0.03	0.03
Schran et al.[3] (2G)	0.04	-

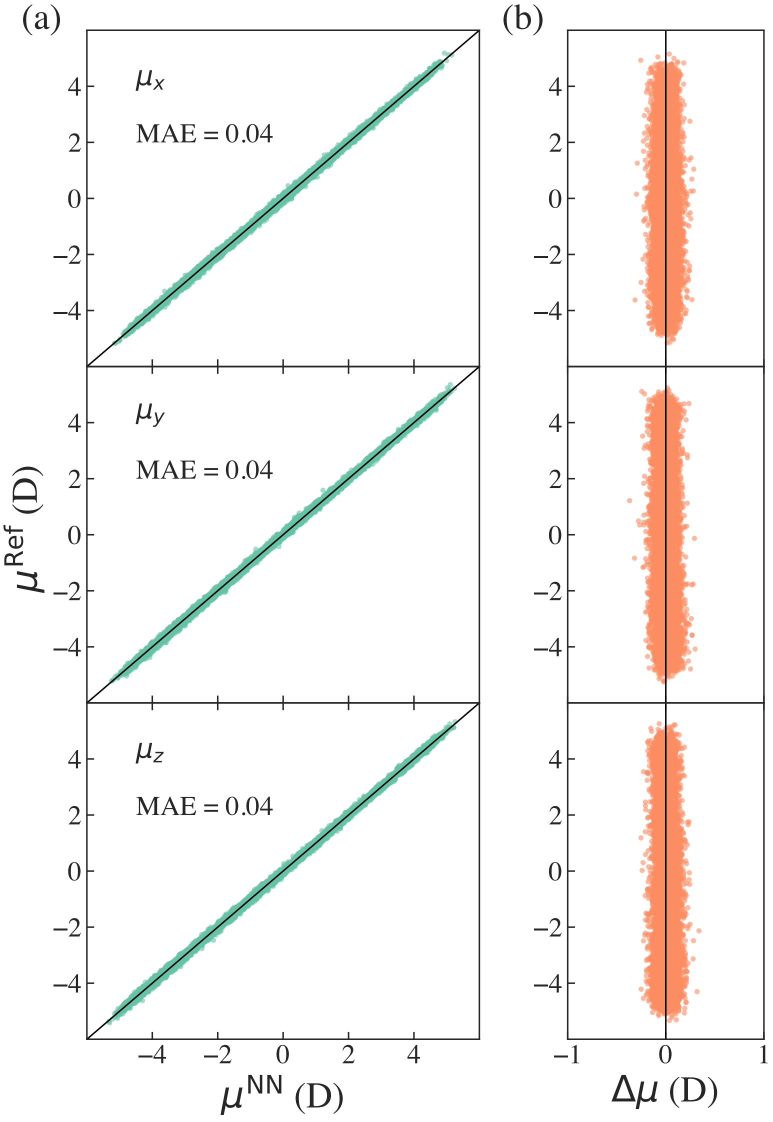

Figure S4: Calculated correlation between the reference and final model predicted dipole moment of bulk PIMD validation set, similar to Fig. 3 in the main text.

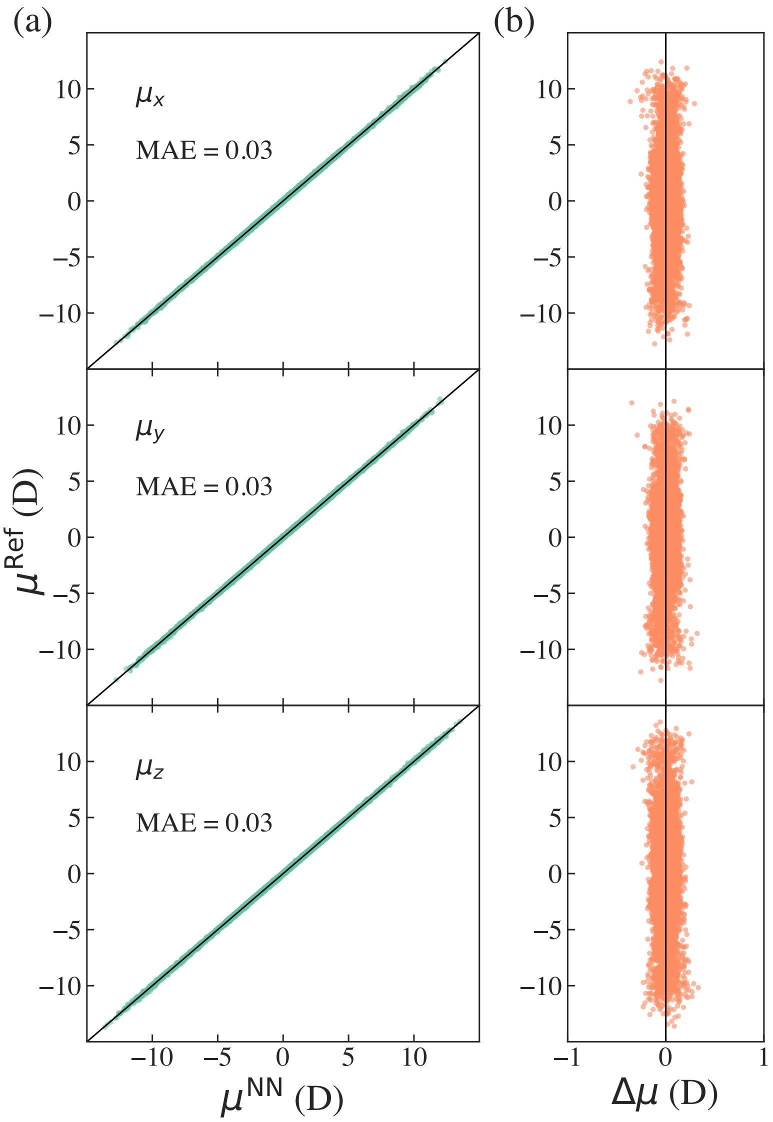

Figure S5: Calculated correlation between the reference and final model predicted dipole moment of the air-water interface classical validation set, similar to Fig. 3 in the main text.

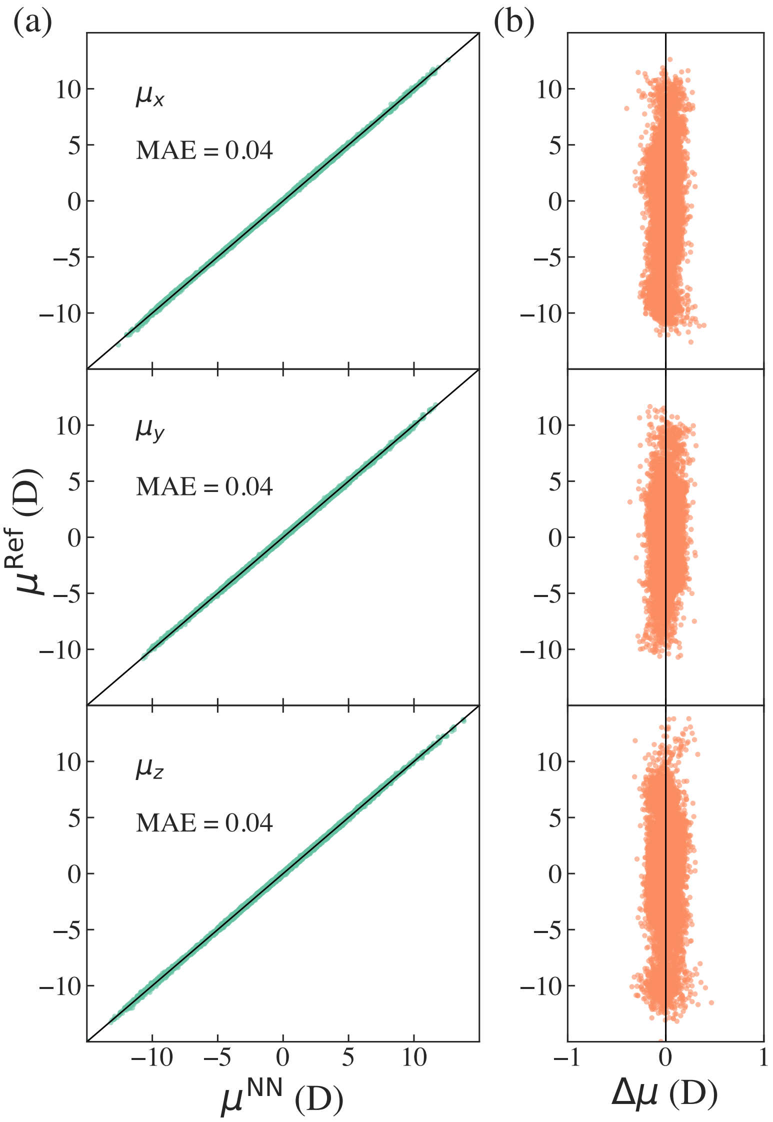

Figure S6: Calculated correlation between the reference and final model predicted dipole moment of the air-water interface PIMD validation set, similar to Fig. 3 in the main text.

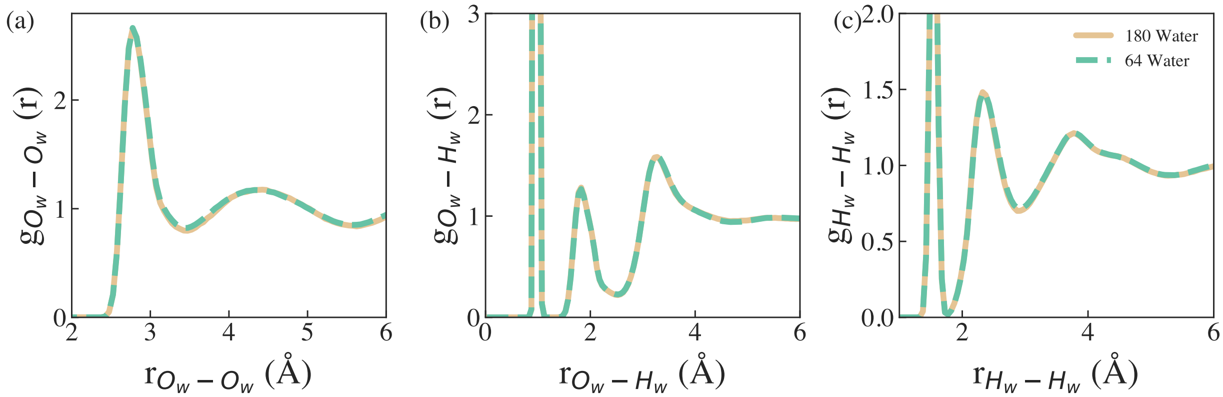

Figure S7: Classical 4G-HDCNNP-calculated RDFs for (a) O_W–O_W (oxygen of water), (b) O_W–H_W (hydrogen of water), and (c) H_W–H_W pairs for simulation boxes containing 180 and 64 water molecules.

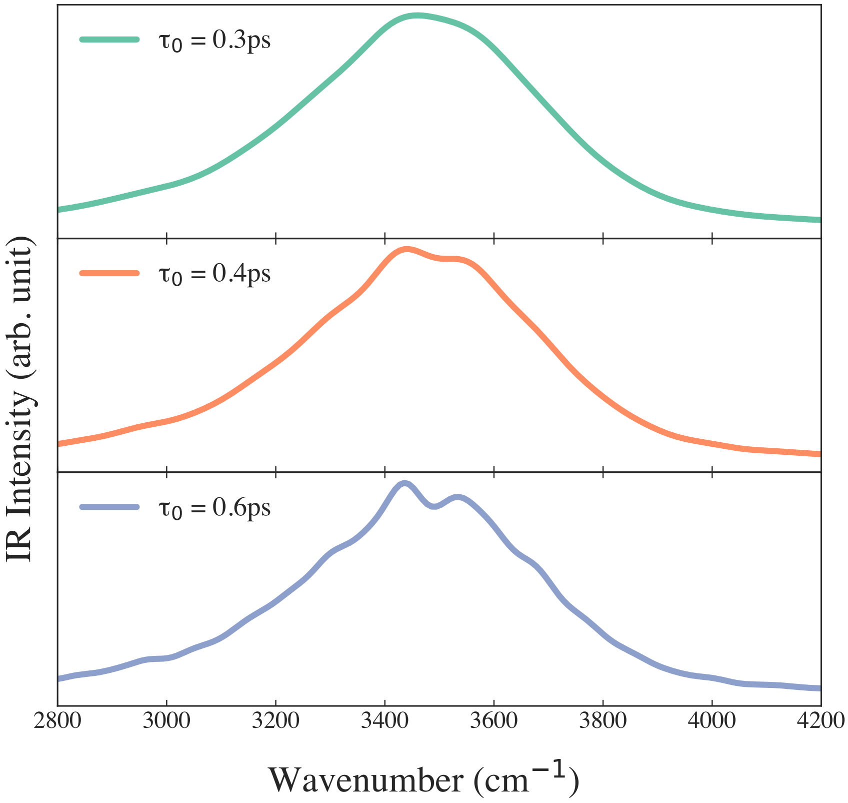

Figure S8: Comparison of the TRPMD calculated IR spectra in the OH-stretch region for bulk water at 298K with Hann window cutoffs of 0.3 ps, 0.4 ps, and 0.6 ps.

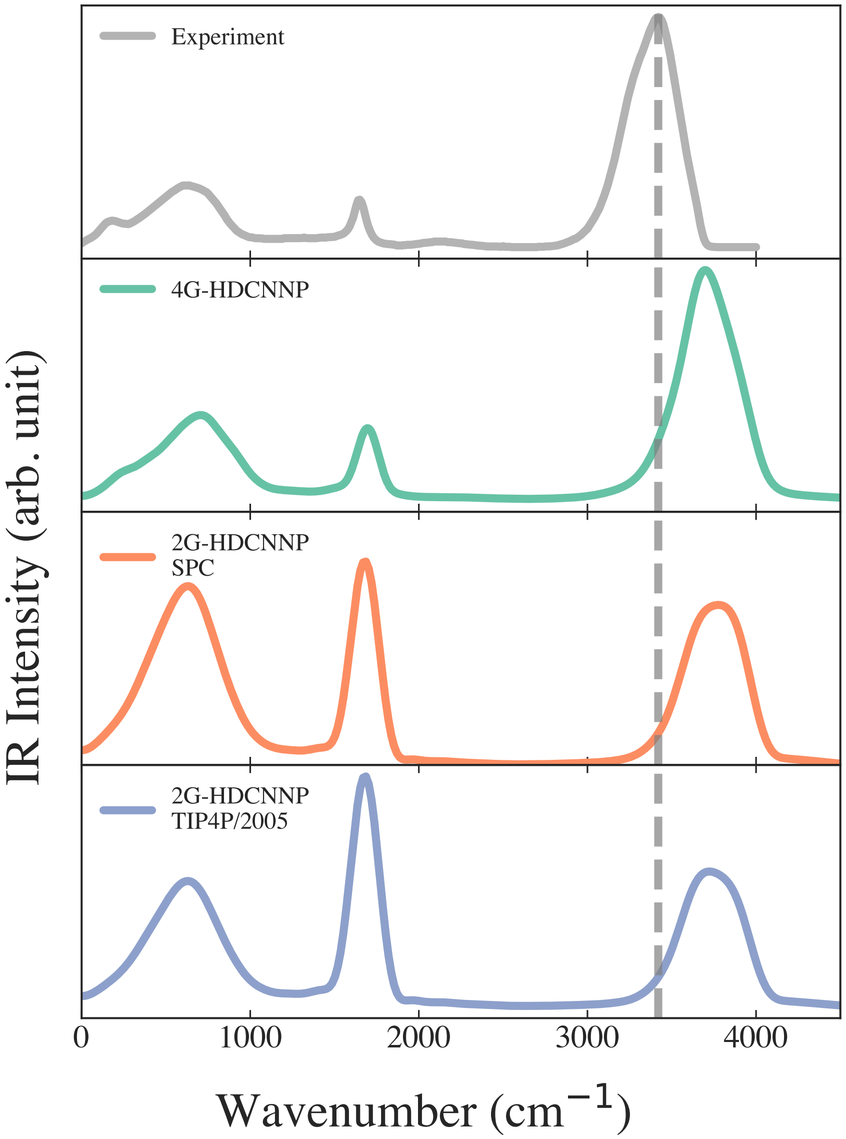

Figure S9: 2G-HDCNNP-calculated classical IR spectra from SPC and TIP4P/2005 charges compared with 4G-HDCNNP and experiment.

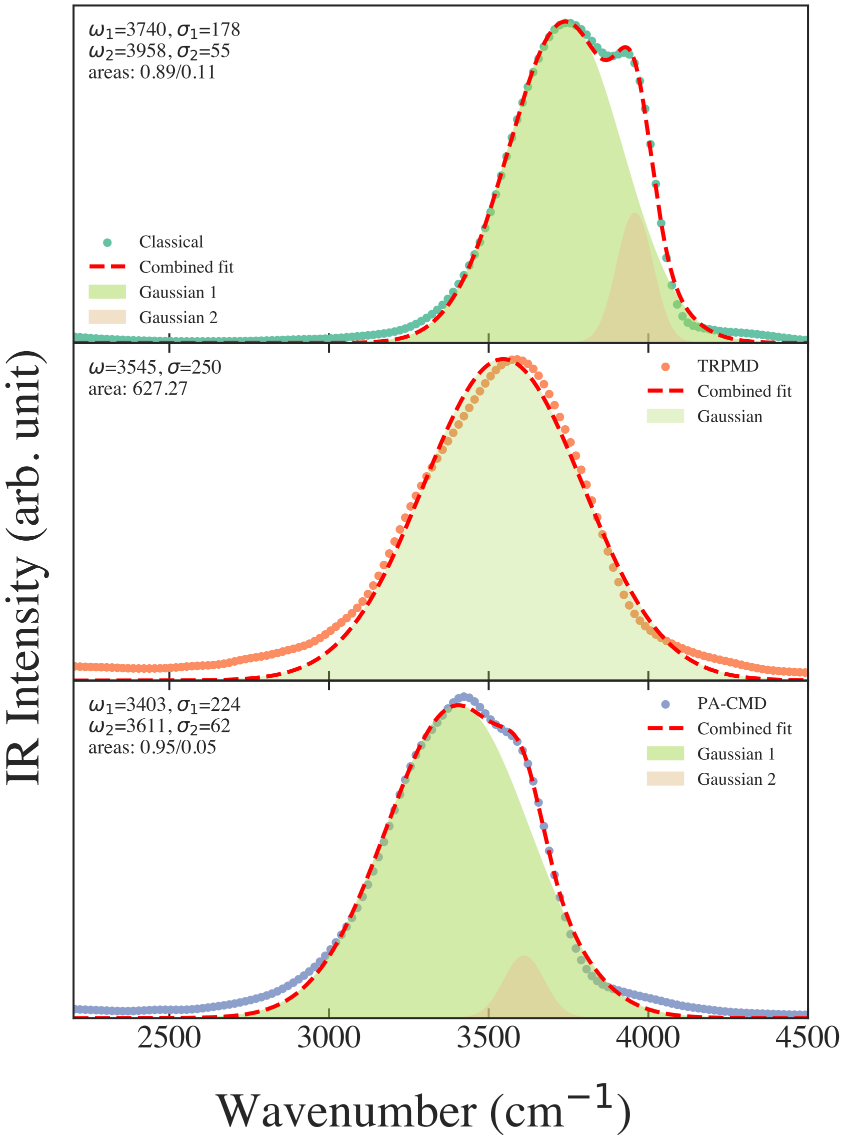

Figure S10: The deconvolution of the O–H stretch peaks of the air-water interface system calculated using different methods.

References

(1) Kocer, E.; Haouari, R. E.; Dellago, C.; Behler, J. Machine Learning Potentials for Redox Chemistry in Solution. *arXiv* **2024**, submitted: 2024-10-04, DOI: 10.48550/arXiv.2410.03299 (accessed 2026-01-07).

(2) Ko, T. W.; Finkler, J. A.; Goedecker, S.; Behler, J. A Fourth-Generation High-Dimensional Neural Network Potential with Accurate Electrostatics Including Non-Local Charge Transfer. *Nat. Commun.* **2021**, *12*, 398.

(3) Schran, C.; Brezina, K.; Marsalek, O. Committee Neural Network Potentials Control Generalization Errors and Enable Active Learning. *J. Chem. Phys.* **2020**, *153*, 104105.
